# Poly (A) binding protein 2 is critical for stem cell differentiation during regeneration in the planarian *Schmidtea mediterranea*

**DOI:** 10.3389/fcell.2024.1433142

**Published:** 2024-09-23

**Authors:** Namita Mukundan, Nivedita Hariharan, Vidyanand Sasidharan, Vairavan Lakshmanan, Dasaradhi Palakodeti, Colin Jamora

**Affiliations:** ^1^ Integrative Chemical Biology (ICB), Institute for Stem Cell Science and Regenerative Medicine, Bangalore, India; ^2^ Manipal Academy of Higher Education, Manipal, India; ^3^ Stowers Institute for Medical Research, Kansas City, MO, United States; ^4^ Department of Life Science, Shiv Nadar Institution of Eminence, Greater Noida, Uttar Pradesh, India

**Keywords:** stem cell, differentiation, poly A binding protein, regeneration, post transcriptional regulation, planaria

## Abstract

Post-transcriptional regulation has emerged as a key mechanism for regulating stem cell renewal and differentiation, which is essential for understanding tissue regeneration and homeostasis. Poly(A)-binding proteins are a family of RNA-binding proteins that play a vital role in post-transcriptional regulation by controlling mRNA stability and protein synthesis. The involvement of poly(A) binding proteins in a wide range of cellular functions is increasingly being investigated. In this study, we used the regenerative model planarian organism *Schmidtea mediterranea* to demonstrate the critical role of poly(A)-binding protein 2 (PABP2) in regulating neoblast maintenance and differentiation. A deficit in PABP2 blocks the transition of neoblasts toward immediate early progenitors, leading to an enhanced pool of non-committed neoblasts and a decreased progenitor population. This is reflected in variations in the transcriptome profile, providing evidence of downregulation in multiple lineages. Thus, an insufficiency of PABP2 resulted in defective formation and organization of tissue, leading to abnormal regeneration. Our study reveals the essential role of PABP2 in regulating genes that mediate stem cell commitment to early progenitors during tissue regeneration.

## Introduction

Highly regenerative planarians are an exceptional model system to elucidate *in vivo* stem cell functions. Their regenerative capability is due to their adult pluripotent stem cell population, called clonogenic neoblasts, that can differentiate into multiple cell lineages (Wagner et al., 2011). Neoblasts respond to injury with a systemic increase in their proliferation rate. In response to tissue loss, a second phase of proliferation occurs locally at the wounded region, which is critical for the formation of undifferentiated tissue known as a “blastema” (Wenemoser and Reddien, 2010). Several regenerative clues such as signals from injury, positional information, and the extracellular matrix are crucial for the expression of cell-type specific differentiation marks (Wenemoser and Reddien, 2010; Scimone et al., 2017; Cote et al., 2019). In planarians, muscle fibers express positional controlling genes in a spatiotemporal manner to provide positional information, helping in stem cell fate determination (Witchley et al., 2013). It has been shown that neoblasts at the S, G2, and M phases of the cell cycle express fate specific transcription factors (FSTF) followed by asymmetric division to form early progenitors (specialized neoblasts) and noncommitted neoblasts (Raz et al., 2021a). Some of the specialized neoblasts can undergo asymmetric division to give rise to daughter neoblasts with non-identical fate-specifying factors (Raz et al., 2021a; Lei et al., 2016). The perturbation of these processes including proliferation and differentiation mediated by asymmetric division or external cues can lead to defective regeneration and tissue turnover.

Studies show that neoblasts undergo massive changes in gene expression during the process of proliferation and differentiation. RNA binding proteins (RBPs) are critical for determining the functionality of transcripts, thereby regulating gene expression. Several genes encoding RNA binding proteins are enriched in the neoblast population, suggesting a prominent role for post-transcriptional regulation in stem cells during planarian regeneration (Krishna et al., 2019; Rouhana et al., 2010; Brais et al., 1999). Post-transcriptional processes such as alternative polyadenylation and poly A tail length modulation govern mRNA stability through intrinsic sequence alterations. Our previous study identified a homolog for *CPSF*, *CsTF*, *CFI*, *CFII*, and the cis sequence binding sites in planarians, critical for polyadenylation and their regulation (Lakshmanan et al., 2016). Polyadenylation at the 3′ UTR of mRNAs has a high affinity for highly conserved RBPs called “poly A binding proteins nuclear” (PABPN) in vertebrates, also known as “poly A binding protein 2” (PABP2) in invertebrates. In mouse models and human cell lines, PABPN mRNA and protein expression levels are lower in skeletal muscles at homeostasis. Interestingly, an increase in PABPN is observed during skeletal muscle regeneration, which suggests a role for this protein during tissue regeneration and repair (Apponi et al., 2013). However, the role of PABP2 in modulating stem cell function during regenerative processes is unknown. PABP2 is a multi-functional protein involved in several molecular processes. For example, it has been shown to interact with RNA polymerase II, suggesting a possible role in transcriptional regulation (Bear et al., 2003). It was also identified as a molecule involved in alternative polyadenylation (De Klerk et al., 2012; Jenal et al., 2012; Martine, 2012), RNA hyperadenylation (Bresson and Conrad, 2013), and the regulation of lncRNAs (Beaulieu et al., 2012). Although the molecular mechanisms of PABPN/PABP2 on RNA have been extensively studied, its impact on stem cell function remains to be defined.

In *Schmidtea mediterranea*, we identified *pabp2* enriched in neoblasts. Here, we report a novel role for PABP2 in stem cell differentiation during tissue regeneration. PABP2 knockdown leads to altered phenotypes in both regenerative and homeostatic conditions. Further analysis showed that PABP2 is critical for stem cell regulation, and its downregulation restricts fate commitment during regenerative processes.

## Materials and methods

### Planarian culture

*Schmidtea mediterranea* (sexual strain- S2F2) were maintained at 18–20°C in an incubator (SANYO MIR 554). The culture was maintained in 1 X Montjuich salt media (1.6 mM NaCl, 1 mM CaCl_2_, 0.1 mM MgCl_2_, 1 mM MgSO_4_, 0.1 mM KCl, and 1.2 mM NaHCO_3_; pH - 7) in milli Q water. The media was filtered using 0.45 μm vacuum filters (membrane filter Millipore 654). Stock animals were fed twice per week with liver paste, and the experimental animals were starved for 7 days prior to the experiment. The experimental animals were maintained in Petri dishes (120 mm) and were cleaned every alternative day.

### RNA extraction

TRIzol (Invitrogen, 15,596,026) reagent was used for RNA extraction. The tissue for RNA extraction was collected in Eppendorf tubes and incubated with 300μL of TRIzol at 80°C for 1 h; one-fifth of the total volume of chloroform was added, mixed by inversion, and kept on ice for 10 min. Subsequently the sample with the reagents was centrifuged at 21,000 g for 20 min at 4°C. The uppermost aqueous layer was collected into a fresh Eppendorf tube without disturbing the interface. An equal amount of pre-chilled isopropanol was added and mixed with the previously collected solution and incubated at −20°C for 30 min. Following incubation, RNA was pelleted at 18,000 g for 20 min at 4°C. The supernatant was discarded, and the pellet was washed twice with 70% ethanol (prepared in nuclease-free water from Invitrogen; code 10,793,837) by centrifuging at 18,000 g for 10 min at 4°C. During RNA extraction from the sorted cells, 1 μL of glycoblue (Invitrogen, AM9515) was added to visualize the RNA pellet. The washed RNA pellet was air dried and reconstituted with nuclease-free water (Invitrogen_10793837) and stored at −80°C.

### cDNA preparation

cDNA was prepared using 1 μg of extracted RNA using a SuperScript II Reverse Transcriptase kit (Invitrogen 18,064,022), oligo (dT) 12–18 Primer (Invitrogen 18,418,012), and RNase OUT Recombinant Ribonuclease Inhibitor (Invitrogen 10,777,019). The reaction was set up as per the protocol manual. The RNA in the reaction mix was digested using RNase H treatment (Invitrogen 18,021,014).

### Gene cloning

Gene specific primers were used to amplify specific genes (*pabp2*: 5′-TAT​GGT​CGA​TAG​CCT​AAA​TGA​CA-3′ and 5′-ATT​ACT​TTT​ATC​TGA​CGA​CTG​CG-3′). LA Taq DNA Polymerase (TaKaRa RR002C) was utilized for PCR amplification of targeted genes. Purified PCR products were inserted into the vector using a TA cloning kit as per their protocol manual (Invitrogen K207040). The ligated products were transformed into competent DH5α cells (*Escherichia coli*) and screened for blue and white colonies under a kanamycin resistant background. Plasmids were extracted from the selected colonies and the sequence confirmed with M13 primers (Invitrogen; N52002 and N53002).

### RNA interference (RNAi)

*pabp2* and *gfp* (control) dsRNA were prepared from gel eluted (Promega, A9282) PCR product using T7 RNA polymerase (NEB, M0251L) as per Buchholz et al. (2006). The product was then treated with DNase-1 (Sigma, 1,014,159,001), and RNA was precipitated with 4 M lithium chloride and 100% ice-cold ethanol. dsRNA synthesis was confirmed by running 1 μL of the purified product on 1.5% agarose gel with 0.5 μg/μL ethidium bromide (Amresco, X328) in TBE buffer (pH - 8.0). The concentration of RNA was assessed by comparing the known nucleic acid concentrations of the DNA ladder bands (NEB, N3232L). The microinjection protocol was followed as mentioned previously (Shibata and Agata, 2018) using Nanoject II injector (Drummond Scientific Company, Broomall, PA, USA, 3–000–204) for prepharyngeal injection of dsRNA. Three shots of 69 nL (stock concentration of dsRNA-1 μg/μL) were administered for three consecutive days followed by a recovery time of 4 days. This regimen was followed, and animals were cut into two (at the prepharyngeal region) on the 11^th^ day and checked for phenotypic defects in a regeneration experiment. In homeostasis, the experiment’s injection regimen was followed for 28–30 days.

### RNAi efficacy assessment

*pabp2* was amplified from 1 µL of cDNA synthesized using 1 µg RNA extracted from control and *pabp2* RNAi samples. Following 25 cycles of PCR amplification, 3 µL of PCR product was mixed with loading dye (NEB, B7021S) and subjected to agarose gel (1.5% agarose prepared in TBE buffer with 0.5 μg/mL EtBr) electrophoresis. Actin was used as a loading control.

### Whole mount immunostaining

Animals treated with 2% HCl and fixed using Carnoy’s fixative (60% ethanol, 30% chloroform, 10% glacial acetic acid); they were stored at −20°C for at least an hour and then rehydrated before being bleached in hydrogen peroxide (20% H_2_O_2_ in methanol). The animals were incubated in blocking solution (10% horse serum in PBSTx) followed by primary antibody (anti arrestin/tmus/anti phospho-histone H3 ser10 (Abcam 47,297)/anti-acetylated tubulin (Sigma) incubation overnight at room temperature or 2–4 h at room temperature at dilutions of 1:5,000 (anti arrestin), 1:100 (tmus), 1:100 (anti phospho-histone 3 ser10), and 1:1,000 (anti-acetylated tubulin). Anti PIWI1 antibody was raised against NEPEGPTETDQSLS antigen in rabbits as described by Guo et al. (2006). The animals were washed with PBS-Tx and stained with secondary antibody (1:400 anti-mouse and rabbit conjugated with both Alexa Fluor 488 and Alexa Fluor 546, molecular probes) before being incubated overnight at 4°C or 2–4 h at room temperature. The animals were then washed and stained with Hoechst (33,342). They were washed, mounted on slides in Mowiol (Sigma, 81,381) containing Dabco (Sigma D2780), and stored in the dark at 4°C.

### RNA probe preparation and fluorescence *in-situ* hybridization

Plasmid with the requisite gene sequence was linearized using NotⅠ or HindⅢ (New England Biolabs) restriction enzyme and was used as template. Digoxigenin-UTP RNA (Sigma, 11277073910) or dinitrophenol-UTP RNA labeling mix (Perkin Elmer NEL555001EA), SP6/T7 (Roche; 10,810,274,001/Invitrogen; AM2718) polymerase along with template was used for riboprobe synthesis, and RNase free DNase (NEB M0303S) was used for template digestion. RNA was purified using Bio-Rad spin mini-column (7,326,830) as per the manufacturer’s instruction. Whole-mount fluorescence *in-situ* hybridization and double fluorescence *in-situ* hybridization was performed as per Pearson et al. (2009) and King and Newmark (2013).

The following primers were used: *agat-1*, 5′-GGA​TTT​CCA​CCG​GTT​TTC​TGT​G-3′ and 5′-AATTGA ACA​CGA​TGT​AAG​CAG​TG-3’; *wi-1*, 5′-CTC​GTT​GGC​AAG​ATT​CAT​CG-3′ and 5′-TGACA CCA​AAT​ACA​AAG​AGA​CA-3’; *h2b*, 5′- TCT​GTT​AAG​AAG​ATT​TCA​AAG​G-3′ and 5′- TCCTGTGTATTTTGTAACAGC-3’; *myoD*, 5′ TCA​ACA​ATA​CCG​ATC​CAG​CCC-3′ and 5′ TCGGG CTTAGCGTCCATTG-3’; *nkx1.1*, 5′ ATTCCAAGTCAAACGATAAGCCT-3′ and 5′ TTCCGTTG GTATTTCTTTAACGG-3’; *pabp2*, 5′-AAT​CAA​TTG​CAT​TTT​TTA​TAT​CT-3′ and 5′-ATCTATC CAA​TTA​TTA​CTC​ATA​A-3’.

### Image acquisition and quantification

An Olympus SX-16 stereomicroscope was used to obtain darkfield images. Confocal images were acquired with an FV 3000 laser scanning microscope (Olympus) with Olympus Flow View software. Image processing and quantification were done using ImageJ software (https://imagej.nih.gov/ij).

Quantification of *wi1* and *pabp2* colocalization was done manually by quantifying the number of *wi1* ^+^ cells and the number of *wi1* ^+^ cells that co-expressed *pabp2*. Among the total *wi1* ^+^ population (899 cells), the percentage of cells that were double-positive for both *wi1* and *pabp2* (244 cells) were calculated. *wi1* and *pabp2* colocalization were quantified from fluorescent *in-situ* hybridization images captured randomly across the whole animal (n = 7) using Fiji software. Blastemal size quantification was done by comparing the blastemal area to the total surface area of the worm (n = 6). Muscle fiber thickness was calculated as an average diameter of three random locations per muscle fiber (total number of fibers measured from a single worm = 30) (n = 5). Protonephridial images were collected dorsally from control and *pabp2* RNAi animals, and the imaging parameters were kept constant. The protonephridial intensity captured per frame was quantified through ImageJ analysis (n = 6). Single fluorescent *in-situ* hybridization was quantified manually by comparing the number of positive cells across total dapi^+^ cells per frame imaged at the blastema (*myoD* (n = 6)*, nkx1.1* (n = 6) and *agat 1* (n = 6) respectively). H3P analysis was done by comparing the total number of positive cells to the whole worm area (i.e., number of H3P^+^ cells/unit area (mm^2^)); (2 dpa, n = 10; 4 dpa, n = 5; 7 dpa, n = 3). For *wi1*/WI1 colocalization analysis, the stem cell population (*wi1* ^+^/WI1^+^) and the immediate mitotic progeny population (WI1^+^) were manually counted using ImageJ at the blastema. The ratio of immediate early progeny to stem cells was calculated among each worm (n = 6). The ratios were compared across the control and *pabp2* RNAi worms.

### Statistical analysis

Statistical analysis was performed used Student’s t-test. An unpaired two-tailed *t*-test was used to check for the *p*-values. All experiments were conducted in biological duplicates, whereas the FACS analysis for mitochondrial potential was conducted in biological triplicates. GraphPad Prism software was used for data analysis.

### Single-cell transcriptome analysis

We used a single-cell transcriptome dataset published from Peter Reddien’s laboratory (Fincher et al., 2018), GEO accession number GSE111764, to extract the cells that express *pabp2*. We used the data matrix submitted in the sequence read archive (SRA) to extract only the cells that express *pabp2*. We reanalyzed the single-cell data as described by Ross and colleagues (BioProject accession number PRJNA432445). We used Seurat (https://satijalab.org/seurat/) to analyze the single-cell transcriptome for the cells that express *pabp2* mRNA (Butler et al., 2018; Stuart et al., 2019). Based on the markers from the single-cell transcriptome dataset (Fincher et al., 2018), we classified the uniform manifold approximation and projection (UMAP) clusters as cell types. We used the “log normalize” method of Seurat on the dataset, which was further scaled (linear transformation) using Seurat. This scaled value was further log-transformed and plotted as a heatmap for genes of interest. We used R ggplot2 GMD and heatmap.2 to derive all the plots.

### Phylogenetic analysis

We aligned the planarian PABP2 sequence with known PABP sequences from other species (obtained from NCBI and GenBank) using MAFFT (version 7.310), and the multiple sequence alignment was visualized using Jalview. The phylogenetic tree was constructed using the IQTree webserver (IQ-TREE 1.6.12). In brief, the ModelFinder functionality implemented within IQTree was used to find the best substitution model for maximum likelihood estimation based on the Bayesian inference criterion (JTT + I + G4). A maximum likelihood tree was then generated using this model, along with bootstrap support (n = 1,000) in iqtree and was visualized using iTOL (iTOL: Interactive Tree of Life (embl.de)). The domain structure of the PABP2 sequence was identified using NCBI CDD.

### Transcriptome analysis

RNA was extracted from the anterior and posterior blastema of the control and *pabp2* RNAi animals at 0–3 dpa. The experiment was conducted in biological duplicates. NEB Next Poly(A) mRNA Magnetic Isolation Module (Catalog number - E7490L) was used for poly A selection, and NEBNext^®^ Ultra™ II Directional RNA Library Preparation with Sample Purification Beads (Catalog number - E7765L) was used for transcriptome library preparation. Sequencing was done using the NovaSeq 6,000 platform using SP flowcell with 2 × 50 bp sequencing read length. All the samples were sequenced in biological replicates. Post sequencing, 11–16 million paired-end (2 * 50 bp) reads were obtained. Adapters were trimmed from the reads using cutadapt v2.10 (-a AGA​TCG​GAA​GAG​CAC​ACG​TCT​GAA​C TCCAGTCA -A AGA​TCG​GAA​GAG​CGT​CGT​GTA​GGG​AAA​GAG​TGT -u 2 -U 2). The trimmed reads were mapped to the *S. mediterranea* transcriptome (Rozanski et al., 2019) using hisat2 v2.1.0 (--rna-strandness R). They (76%–80% mapping percentage) were counted using feature Counts v2.0.0. DESeq2 v1.40.1 was used to perform the read count normalization and differential expression analysis. The plots were generated in R v4.3.0. We have considered a log_2_FC threshold of 0.58 and an adjusted *p*-value (q) of less than 0.05 for classifying a gene as differentially expressed. Different planarian cell-type markers were obtained from the available single-cell transcriptome data (Fincher et al., 2018). The sequencing data reported in this manuscript have been deposited at NCBI- Sequence Read Archive (SRA), project ID: PRJNA1031933.

### Fluorescence activated cell sorting

Cell suspension for FACS sorting was prepared as per Mohamed et al. (2021). The worms (n = 30) were macerated in calcium and magnesium-free buffer (CMFB) and mechanically sheared using a micropipette and strained using 70 μm cell strainers. The cells were centrifuged at 300 × g for 10 min, and the resulting single-cell suspension was stained with Hoechst 33,342 (40 μg/mL) for 40 min. Each subgroup among the control and *pabp2* RNAi was FCCP treated (10 μM for 10 min). All samples were stained with rhodamine 123 (2 μM for 10 min) and analyzed using a BD Fortessa flow cytometer. The mitochondrial potential was assessed in control and *pabp2* RNAi separately by calculating the decrease in potential in comparison with the FCCP-treated subgroups.

## Results

### *pabp2* is enriched in the neoblast and epidermal cell populations

Previously, E2FB12/PABPC1 and PABPC2 were identified in *S. mediterranea* (Wang et al., 2010; Bansal et al., 2017). Here, the newly identified PABP2 sequence (dd_Smed_v6_21160_0_1) is clustered into a branch of PABP2 sequences from other species (Supplementary Figure S1A); it is evolutionarily distinct from the previously known PABPC. Domain analysis revealed the presence of a single RNA recognition motif (RRM) which is a feature of PABPN (Supplementary Figure S2B). Furthermore, the PABP2 sequence shows similarity with the PABPN sequences of various organisms in multiple sequence alignment (Supplementary Figure S1C). Thus, the identified *pabp2* is presumed to be a *pabp2* homologous gene in *S. mediterranea*.

**FIGURE 1:**
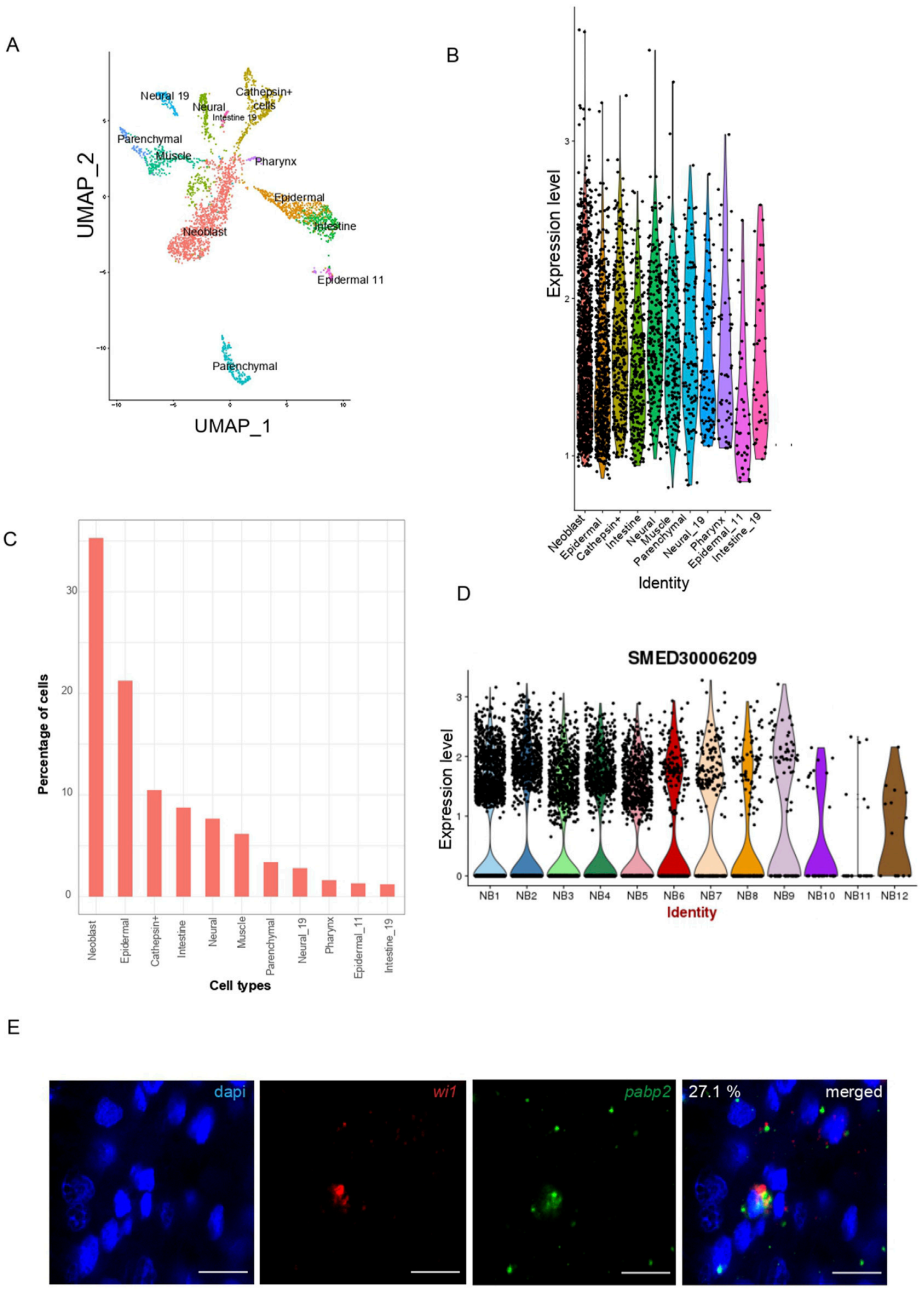
Characterization of *pabp2*. **(A)** UMAP plot obtained from single-cell transcriptome data (Fincher et al., 2018) showing *pabp2* distribution across different cell types. **(B)** Violin plot depicting the expression level of *pabp2* across different cell types. **(C)** Percentage of cells expressing *pabp2* among different cell types. **(D)** Violin plots showing the distribution of *pabp2* across 12 neoblast clusters. **(E)** Fluorescent *in-situ* hybridization to study the expression pattern of *pabp2*. Double fluorescent *in-situ* hybridization showing colocalization of *pabp2* with *wi1*. 27.1% of *wi1* ^+^ population co-expressed *pabp2*, i.e., among 899 *wi1* expressing cells, 244 co-expressed *pabp2*. Probes are indicated (*wi1*-red, *pabp2* - green), scale bars, 10 μm (n = 7).

We investigated the expression of *pabp2* across different cell types using single-cell transcriptome data (https://radiant.wi.mit.edu/app/) and noticed the predominant expression of *pabp2* in neoblasts (35%), followed by the major epidermal cluster (21%), cathepsin^+^ cells (11%), and the intestinal cell population (8%) (Figures 1A–C). The *in-vivo* expression of *pabp2* in neoblasts was confirmed through double fluorescent *in-situ* hybridization using *wi1* (pan neoblast marker) and *pabp2* (Figure 1E). This analysis revealed that 27% of the neoblast population co-expressed *pabp2*, which is consistent with the single-cell data revealing 35% of neoblast co-expressing *pabp2*. From single cell RNA sequencing on neoblast enriched for both high wi1 transcript and protein expression, neoblasts have been classified into 12 different classes (Nb1-Nb12) (Zeng et al., 2018). Among these classes, the *tspan1* ^*+*^ population (Nb2) was shown to exhibit clonogenic properties (Zeng et al., 2018). Some 35% of *tspan1* ^*+*^ neoblasts, which co-express *tgs1*, was shown to be neuronal specialized neoblasts (Fincher et al., 2018). Expression of *pabp2* was notably high in *tspan* ^*+*^ neoblasts, suggesting a crucial role for PABP2 at stem cell regulation (Figure 1D). The second-highest enrichment of *pabp2* was found in the epidermal cluster. The maintenance of epidermal integrity is imperative for wound healing and regeneration in planarians (Bansal et al., 2017). Altogether, *pabp2* was predominantly expressed in neoblasts and the epidermis, suggesting a role for the protein in stem cell function and regeneration.

### *pabp2* is an essential regulator of planarian regeneration and homeostasis

**FIGURE 2:**
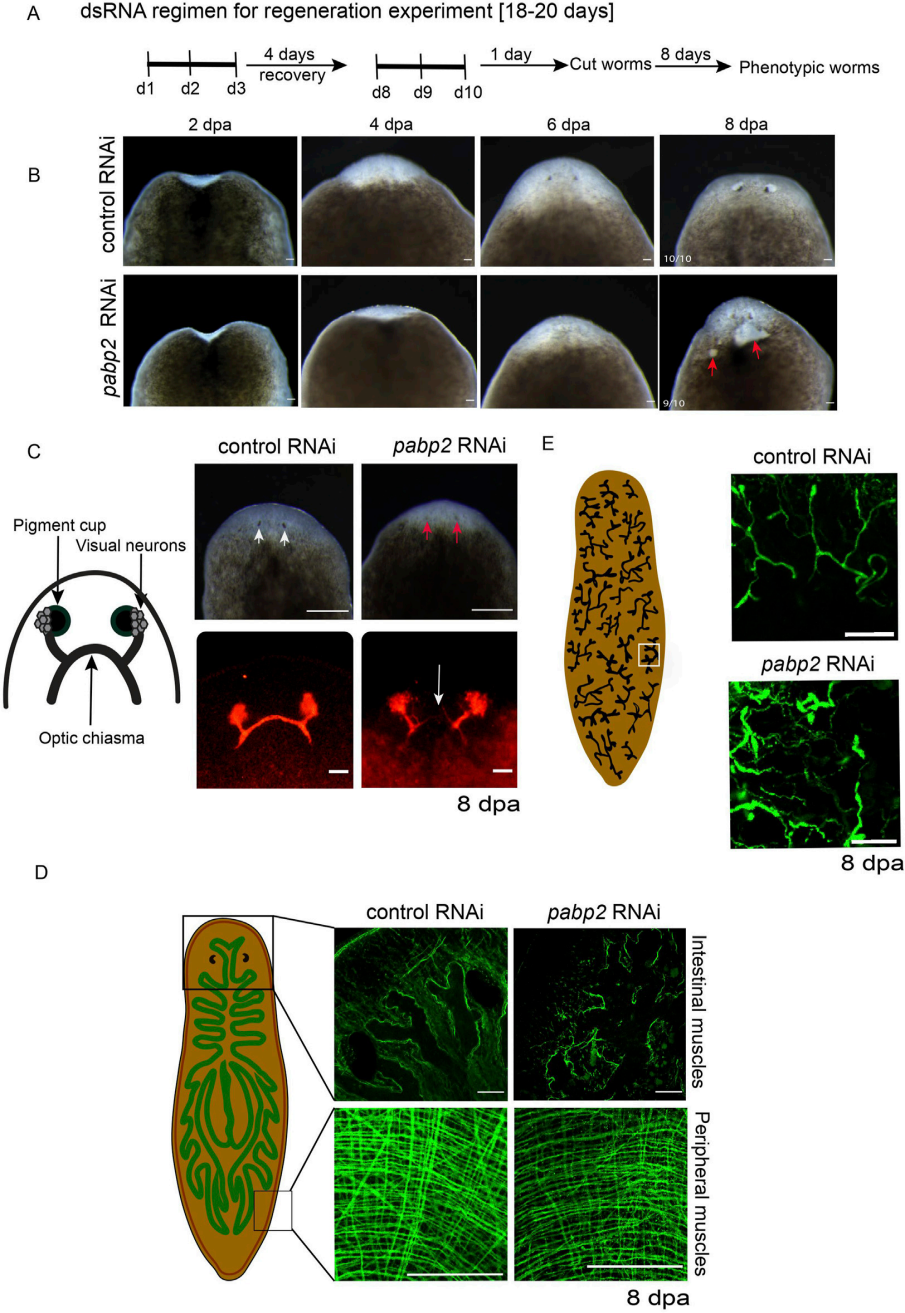
**(A)** Schematic representation showing the timeline of dsRNA administration for the regenerative RNAi experiment. **(B)** Representative images captured on 2, 4, 6, and 8 dpa. The red arrow points toward the lesions developed on the *pabp2* RNAi animals during the course of regeneration (n = 10), scale bar, 200 μm. **(C)** Schematic representation representing the organization of photoreceptors, visual neurons, and optic chiasma in the planarian photoreceptor. Image of animals with photoreceptor where phenotypic animals exhibit underdeveloped eyes. White arrows indicate the photoreceptor pigment in control, and the red arrow represents the reduction in the photoreceptor pigment in *pabp2* RNAi, scale bar, 200 μm. Confocal image of anti-arrestin immunostaining staining for photoreceptor neurons at 8 dpa. The white arrow represents lack of optic chiasma formation in *pabp2* RNAi (n = 5), scale bar, 50 μm. **(D)** Schematic representation of peripheral and intestinal muscle fiber organization in planarian. Confocal image of whole mount tmus immunostaining to visualize the organization of anterior intestinal and peripheral muscle fibers at 8 dpa (n = 5), scale bar, 50 μm. **(E)** Schematic representation of the arrangement of protonephridia embedded in the epithelial tissue. Marked in the white box is a single protonephridia unit. Confocal image of anti-acetylated tubulin immunostaining showing organization of protonephridia in control RNAi and *pabp2* RNAi at 8 dpa (n = 6), scale bar, 50 μm.

Previously, *pabpc1* and *pabpc2* were shown to have a role in meiotic progression and epidermal integrity, respectively (Wang et al., 2010; Bansal et al., 2017). To discern whether PABP2 has any functional significance during planarian regeneration and homeostasis, we knocked down *pabp2* expression. RNAi based knockdown was done through microinjecting *pabp2* or *gfp* (control) dsRNA for three consecutive days, followed by a 4-day recovery period. Following two rounds of injections, the animals were amputated for the regeneration experiment, and we observed that the *pabp2* RNAi animals exhibited body lesions and underdeveloped photoreceptors (Figures 2A,B); they were subsequently lysed by day 20 of the injection regimen. The efficacy of *pabp2* RNAi was assessed by PCR amplification (Supplementary Figure S2B). Phenotypes were observed in 90% of animals by 8 dpa (days post amputation). Blastema size quantification at various time points during regeneration revealed a significant (∼37.5%) reduction by 7 dpa at both anterior and posterior regenerating fragments (Supplementary Figure S2A). Under homeostatic conditions, *pabp2* RNAi animals displayed head regression and had lesions on their body (Supplementary Figure S2C, D). Of the animals, 71% exhibited these phenotypes and underwent lysis within 30 days of the injection regimen. In summary, PABP2 is necessary for tissue regeneration as well as homeostasis.

### *pabp2* knockdown impairs terminally differentiated tissues

The gross phenotypes in *pabp2* RNAi are the stunted blastema growth and lesions in the regenerating animals, suggesting a defect in the blastema and the epidermal tissue. We further focused on delineating the effect of *pabp2* RNAi on various tissues during the regenerative processes. At 8 dpa, animals were collected and experimentally assessed for regenerative defects. The planarian photoreceptor consists of pigment cups, photoreceptor neurons (PRN) and the axonal bundle arising from the dorsally located cell body which joins together to form the optic chiasma (Macrae, 1964; Agata et al., 1998; Shettigar et al., 2017). Phenotypic animals were observed with reduced photoreceptor pigmentation, suggesting a possible defect in eye regeneration (Figure 2C). We investigated the organization of photoreceptors through VC1 immunostaining and observed a lack of optic chiasma formation (Figure 2C). Previous studies reported mutations in PABP2 leading to oculopharyngeal muscular dystrophy patients (Brais et al., 1999; Brais et al., 1998). In planarians, tissues are surrounded by muscle fibers which provide support and also express positional control genes (PCG) critical for regeneration. Upon injury, the subepidermal muscle fibers provide positional cues essential for tissue regeneration (Witchley et al., 2013). We thus examined the possibility that an impaired muscle fiber organization may be responsible for the defective optic chiasma formation. Using tmus immunostaining, we observed that pabp2 RNAi perturbs the organization of both peripheral and intestinal muscles (Figure 2D). Planarian subepidermal myofibers are arranged in four different layers: outer circular, longitudinal, diagonal, and inner longitudinal (Cebrià et al., 1997). We found a 33% reduction in myofiber thickness upon quantification of circular muscle fiber thickness at 8 dpa (Supplementary Figure S2E). Since *pabp2* RNAi leads to defective muscle organization, we also examined the organization of other tissues such as protonephridia, which is embedded in epithelial tissue and consists of multiple cell types that are organized to form a branched pattern (Rink et al., 2011) at 8 dpa. Anti-acetylated tubulin immunostaining revealed a disorganized protonephridial branching pattern (Figure 2E). Protonephridial intensity increased by 33%, indicating a spread of protonephridial organization (Supplementary Figure S2F). Taken together, we observed defects in terminally differentiated tissue, resulting either from an intrinsic neoblast defect failing to replace cells during regeneration or defective muscle fibers, which could lead to disorganized organ systems.

### Knockdown of *pabp2* leads to increased neoblasts at the blastema

**FIGURE 3:**
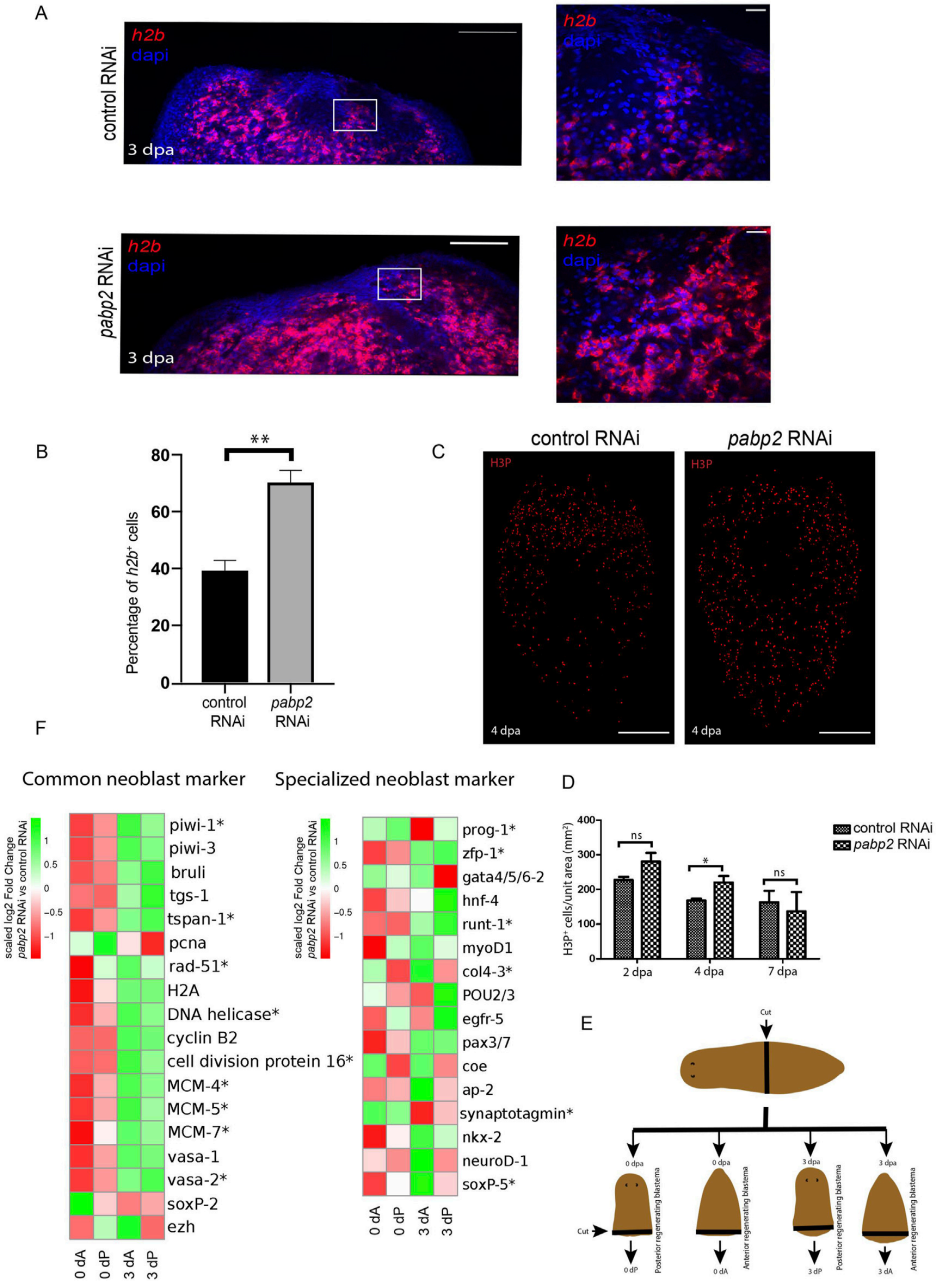
**(A)** Confocal images of fluorescent *in-situ* hybridization at the regenerating surface for *h2b* among control RNAi and *pabp2* RNAi (scale bar, 100 μm) and the zoomed images (scale bar, 20 μm). White boxes indicate the magnified area in the zoomed images in the columns to the right. **(B)** Quantification of the percentage of *h2b* ^+^ cells (n = 6) from the zoomed images, **p < 0.01. **(C)** Maximum intensity projections of confocal images showing H3PS10^+^ cells at 4 dpa among control RNAi and *pabp2* RNAi (scale bar, 50 μm). **(D)** Quantification of the total number of mitotic cells (H3PS10^+^) at 2 dpa (n = 8), 4 dpa (n = 5, and 7 dpa (n = 3), *p < 0.05; n.s. *p* > 0.05. Number of H3PS10^+^ cells counted at 2 dpa (control RNAi- 4,188, *pabp2* RNAi- 8,052), 4 dpa (control RNAi- 2,283, *pabp2* RNAi- 4,475), and 7 dpa (control RNAi- 502, *pabp2* RNAi- 572). **(E)** Schematic representation showing the methodology followed during sample collection for transcriptome analysis across control and *pabp2* RNAi. The RNAi animals were cut at the prepharyngeal region into anterior regenerating and posterior regenerating animals. Blastema was collected at 0 and 3 dpa from anterior (0 dA, 3 dA) and posterior (0 dP, 3 dP) regenerating animals, respectively. **(F)** Heatmap depicting the expression of stem cell-specific markers at 0 and 3 dpa across anterior (0 dA, 3 dA) and posterior (0 dP, 3 dP) regenerating blastema, respectively. The asterisk indicates that at least one of the four comparisons included in the heatmap is statistically significance (q < 0.05) for that gene. Details about the fold change and the normalized read counts are included in Supplementary Tables S1, S2.

Since *pabp2* is enriched in the neoblast population, its depletion could either lead to a defect in proliferation, differentiation, or both. One of the possibilities is depletion of the neoblast population subsequently resulting in decreased progenitors. A second possibility could be an intrinsically defective neoblast population in *pabp2* RNAi animals, affecting the later stages of differentiation. Thus, we checked the stem cell population by probing for *h2b* ^+^ and *wi1* ^+^ cells (neoblast markers) using whole mount fluorescent *in-situ* hybridization at 3 dpa (Figure 3A; Supplementary Figure S3A). We found a significant (*p* < 0.05) increase in *h2b* ^+^ and *wi1* ^+^ cells in *pabp2* RNAi animals, suggesting an overall increase in the stem cell pool. Furthermore, we checked for mitotic neoblast (cells at G2 to M phase) using antibody against H3PS10 on 2, 4, and 7 dpa. We observed a slight increase (1.3 fold, *p* < 0.05) in the H3P^+^ population at 4 dpa, but no change in the numbers was observed at 2 dpa. To ensure that no major changes occurred in the mitotic cell numbers at later time points of regeneration, we left a few knockdown animals until 7 dpa and measured the number of H3P^+^ cells. We did not notice any observable changes in the H3P^+^ cells, suggesting that the *pabp2* RNAi has no effect on the mitotic phase of the neoblast population (Figures 3C,D). Together, our data suggest that neoblast maintenance was not affected in the *pabp2* RNAi animals. It is possible that the increased neoblast could impair differentiation, thereby leading to accumulation of unspecified neoblasts. However, the function of PABP2 in neoblast differentiation requires further investigation.

To further study the effect of *pabp2* RNAi on neoblast maintenance, we conducted transcriptome profiling of 0 dpa and 3 dpa blastema from control and *pabp2* RNAi animals. At 3 dpa, we observed a significant upregulation of neoblast-specific transcripts including *wi1*, *tspan*, *vasa*, and cell cycle proteins like *DNA helicase* and *pcna* (Figure 3E; Supplementary Table S1), which correlates with fluorescent *in-situ* hybridization experiments, suggesting an increased neoblast pool. Planarian neoblasts are a heterogeneous group consisting of distinct subpopulations known as “specialized neoblasts”. An extensive single-cell transcriptome analysis from the X1 population of neoblasts (dividing neoblasts) identified 14 different clusters of neoblasts, with neoblast cluster 2 revealing clonogenic properties. Further analysis also showed TSPAN as a surface marker expressed on the clonogenic neoblast. Another study showed that *tspan* ^+^ neoblast co-expresses *tgs-1*, which also specifies neuronal specialized neoblasts (Fincher et al., 2018). Our transcriptome analysis showed increased expression of *tspan* in the *pabp2* RNAi animals, suggesting the enrichment of clonogenic neoblasts. However, based on Fincher et al. (2018), we cannot also rule out that the neoblasts express neuronal lineages. It has been shown that the expression of fate-specific transcription factor (FSTF) regulates neoblast fate, and a neoblast can asymmetrically divide to give rise to fate-specified progeny (FSTF^+^) and a pluripotent neoblast (FSTF^−^) (Raz et al., 2021a). *zfp-1*, a FSTF expressed by epidermal specialized neoblasts, is essential to maintaining the epidermal lineage. Low levels of *zfp1* expression in the intestinal neoblast coincide with endocyte-specific markers (King et al., 2024). *zfp-1* was upregulated in *pabp2* RNAi animals (Figure 3E; Supplementary Tables S2, S3), suggesting a possible defect in the epidermal or intestinal lineages. In addition, we also analyzed the transcriptome data for the expression of the early lineage markers. Most of the early lineage markers, such as *ap2*, *coe*, *ston2* (neuronal marker), *hnf4*, and *gata4/5/6* (intestinal marker), did not show any significant difference in expression (*p* > 0.05) or downregulation (*p* > 0.05) in the *pabp2* RNAi animals (Figure 3E; Supplementary Table S2). In summary, our transcriptome data from the *pabp2* RNAi animals showed upregulation of the global stem cell specific markers.

### PABP2 is critical for cellular differentiation

**FIGURE 4:**
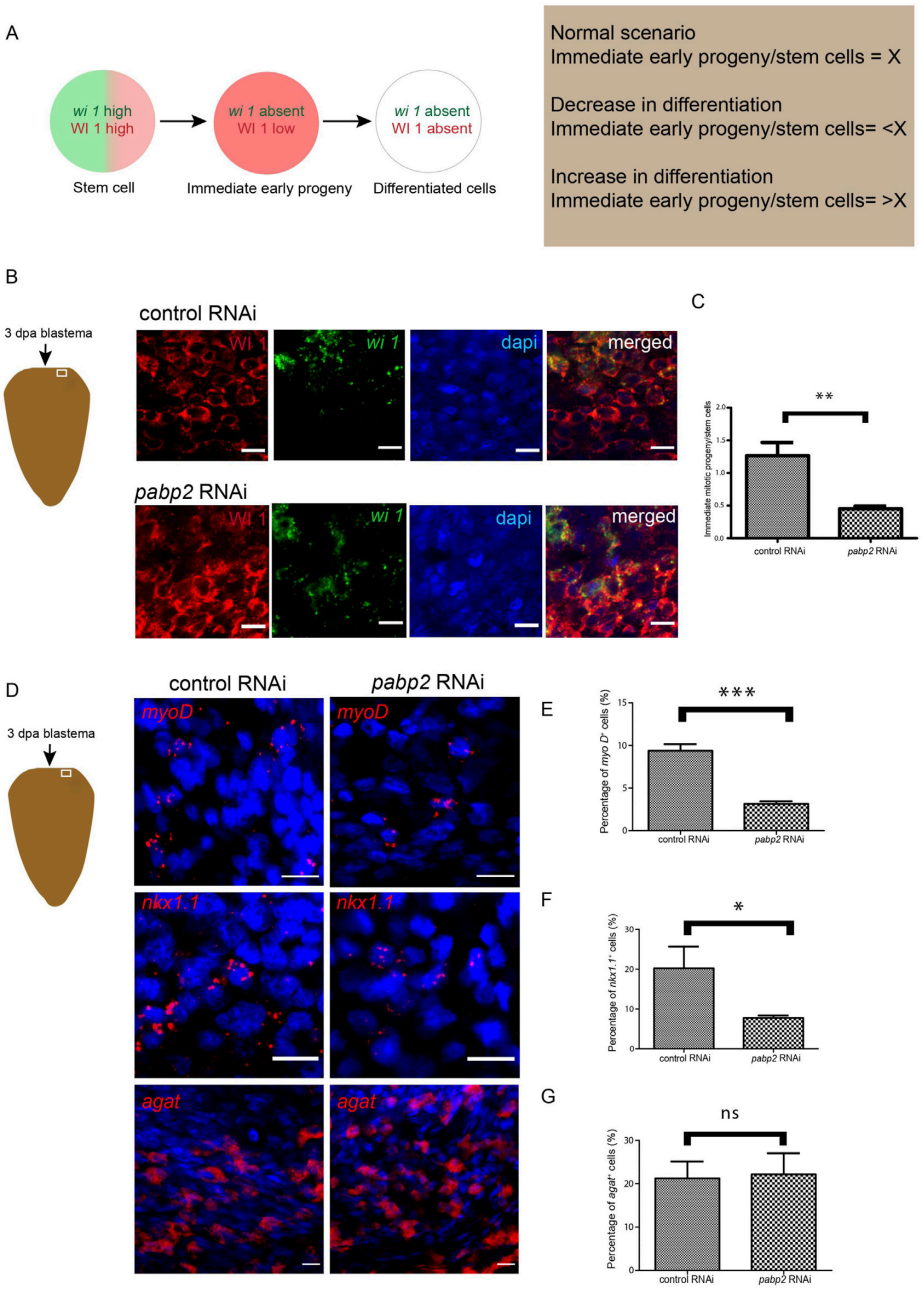
**(A)** Schematic representation showing the procedure adapted for quantifying differentiation defects. Green and red denote the presence of wi1 transcript and protein, respectively. Ratio of immediate early progeny to stem cell reflecting the variations in differentiation. **(B)** Schematic representation indicates the area of imaging at 3 dpa blastema. Confocal imaging of colocalization of *wi1* (green) and WI1 (red) using fluorescent *in-situ* hybridization and immunofluorescence, respectively, scale bar, 10 μm. **(C)** Graphical representation of decrease in differentiation. Graph plotted from the quantified ratio of immediate early progeny to stem cell population (n = 6), **p < 0.01. **(D)** Fluorescent *in-situ* hybridization probing for various progenitor populations and its quantifications (*myoD* - longitudinal muscle fiber, *nkx1.1* - circular muscle fiber, *agat 1* - epidermal cells) across control RNAi and *pabp2* RNAi, scale bar, 10 μm. **(E–G)** Representation of percentage of progenitor-expressing cells compared across control and *pabp2* RNAi for *myoD* (n = 5), *nkx1.1* (n = 6) and agat 1 (n = 6), respectively. *p < 0.05, ***p < 0.001, n.s. *p* > 0.05.

Overall, an increase in neoblast number accompanied by defects in terminally differentiated tissue could result from defective commitment of neoblasts toward specific lineages (Cebrià et al., 2018). In order to determine if there is a differentiation defect in the *pabp2* RNAi scenario, a colocalization experiment probing for wi1 (fluorescent *in-situ* hybridization for mRNA) and WI 1 (immunostaining for protein) was conducted. *wi1* transcript is expressed in the neoblast whereas the corresponding protein is enriched both in the neoblast and the progenitors (Guo et al., 2006) (Supplementary Figure S4A). Stem cells express both *wi1* mRNA and protein, and the WI1 protein endures for 72 h in post-mitotic cells (Guo et al., 2006). wi1 (−)/WI1 (+) cells represent the transition state from stem cell to postmitotic progeny, meaning that they mark the immediate early progeny. A change in the differentiation process can be measured by calculating the ratio of immediate early progeny with the stem cell population. An increase or decrease in this ratio will denote an increased or decreased differentiation process. Interestingly, the ratio at 3 dpa was 1.33 in control animals and decreased to 0.45 in *pabp2* RNAi animals, thereby indicating a significant decrease in the immediate early progeny (Figures 4B,C). Taken together, these data demonstrate that a decrease in differentiation is reflected by an increase in the stem cell population.

It has been shown that neoblasts express fate-specifying transcription factors required for their transition toward respective progenitors (Scimone et al., 2014). To decipher how the progenitor populations are affected, we performed fluorescent *in-situ* hybridization by probing for markers such as *myoD* and *nkx1.1* which mark the longitudinal and circular muscle fibers respectively, and found a 60% decrease in *myoD*- and 50% decrease in *nkx1.1*-expressed cells (Figures 4E,F), corroborating the observed muscle fiber defect. Lineage specification during the epidermal differentiation process is very well studied in planarians. It is also known that the zeta neoblast lineage gives rise to epidermal tissue and that agat 1 marks the latter epidermal progenitors (Wolfswinkel et al., 2014; Tu et al., 2015). Since we found an enrichment of *pabp2* in the epidermis (Figure 1), we quantified the epidermal progenitor population. However, there is no change in the *agat 1* ^+^ epidermal progeny in *pabp2* RNAi animals (Figure 4G). This also corroborates our previous finding that the *pabp2* RNAi animals showed increased expression of *zfp1*—a zeta neoblast marker. The lesions observed could be due to the faulty body wall muscle organization, which was evident from muscle staining in the *pabp2* RNAi animals (Figure 2D). Altogether, we found a decrease of *myoD-* and *nkx 1.1*-expressing cells, whereas *agat 1* ^+^ cells remain unchanged, suggesting a possibility of muscle lineages being downregulated. To conclusively understand the effect of *pabp2* RNAi on other lineages, it is essential to study the expression of a wide variety of lineage-specific markers.

### PABP2 regulates transcripts essential for major epidermal population and intestinal lineages

**FIGURE 5:**
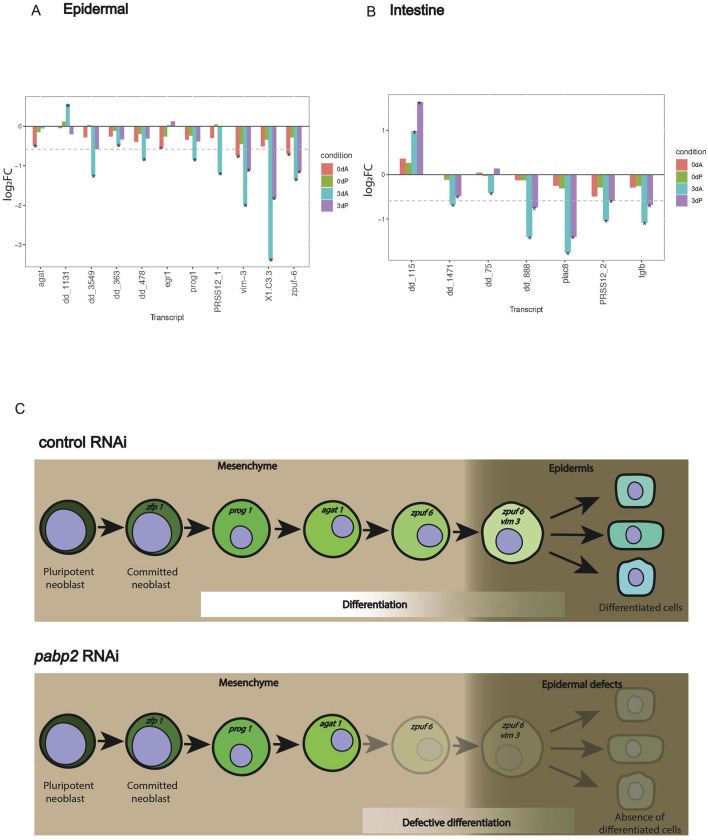
**(A, B)** Bar plot showing gene expression variation among specific lineages. Transcripts with variations among **(A)** epidermal and **(B)** intestinal markers compared between *pabp2* RNAi and control RNAi at various time points (as denoted by color bars within plots) among anterior and posterior regenerating blastema. The comparison is between *pabp2* RNAi versus control RNAi in each of the condition denoted by the bar color within the plots. Transcript PRSS12_1 (dd_Smed_v6_351_0_1) is specific to epidermis, and PRSS12_2 (dd_Smed_v6_790_0_1) is specific to intestine. The asterisk indicates statistical significance (q < 0.05), and the dotted line on y-axis represents log2FC −0.58. All cell-type specific transcripts with q < 0.05 in at least one of the four comparisons are included in the graph. Details about fold change and normalized read counts are provided in Supplementary Table S3. **(C)** Working model depicting the critical role of PABP2 in epidermal differentiation. The epidermal markers from the lineage progression during its transition from mesenchyme to epidermis across control and pabp2 RNAi. *pabp2* RNAi leads to defective differentiation post-*agat 1* stage.

Transcriptome analysis was performed to understand the effect of *pabp2* RNAi on commitment toward various cell lineages. It has been reported that most lineage specification marks are expressed by 3 dpa (Wenemoser and Reddien, 2010). Transcriptome sequencing was conducted at 0 dpa and 3 dpa blastema in control and *pabp2* RNAi animals to investigate the expression of different cell-type markers (Fincher et al., 2018). Among the various cell types, transcripts representing major epidermal clusters were drastically downregulated, such as *egr 5*, *zpuf 6*, and *vim 3* by 3 dpa (Figure 5A; Supplementary Table S3), which are critical for later stages of epidermal differentiation (Tu et al., 2015). At 3 dpa, there was a minor change in *prog* expression (log_2_FC −0.83 and −0.38, respectively, in the anterior and posterior regenerating blastema). In the anterior and posterior regenerating blastema, we did not observe a reduction in *agat 1* expression at 3 dpa (log_2_FC −0.05 and −0.01), and our fluorescent *in-situ* hybridization showed no change in *agat 1* ^*+*^ cell population (Figure 4D). Together, our results suggest that epidermal lineage defects in *pabp2* RNAi are caused by defects in later stages of differentiation among epidermal cell types rather than early progenitors.

Our analysis revealed a drastic decrease in intestinal-specific transcripts across anterior and posterior developing blastema (Figures 3E, 5B). The *cathepsin* ^+^ cluster consists of a heterogeneous group of cells which includes pigment cells, glial cells, and several other unknown cell types (Lucila et al., 2018). *Cela1* and *jag1* specific for *cathepsin* ^+^ cells showed downregulation at 3 dpa (Supplementary Figure S5A, Supplementary Table S3), suggesting that among the mixed population of *cathepsin* ^+^ cells, a few subtypes were affected upon *pabp2* RNAi. In the posterior developing blastema, we also observed few transcripts being downregulated among the pharyngeal cluster (Supplementary Figure S5B, Supplementary Table S3). Furthermore, we also observed few muscle-specific transcripts being downregulated which could possibly result from an indirect effect of suboptimal levels of *pabp*2 (Supplementary Figure S5C, Supplementary Table S3). The cell types which did not show significant variation include the parapharyngeal, neuronal, ciliated neuronal, and non-ciliated neuronal lineages upon *pabp2* RNAi (Supplementary Figure S5D, S5E, S5F, S5G, Supplementary Table S3). In summary, our bulk transcriptome data from the control and *pabp2* RNAi animals overlaid on the existing single cell data showed defects predominantly in the major epidermal population, a subset of cathepsin^+^ cell types and intestinal lineage.

## Discussion

Our study revealed a role for PABP2 in the transition of stem cells to differentiated cell types. PABP2 insufficiency leads to enhanced self-renewal of intrinsically defective neoblasts and dysregulated differentiation. Hence, a defect in PABP2 functioning affects the process of differentiation, thereby leading to an insufficient progenitor population resulting in improper tissue regeneration and organization.

Cellular differentiation is the process by which committed cells undergo drastic gene expression changes to become specialized cells. In humans, defective differentiation can lead to several disease conditions affecting specific cell lineages, such as muscle (Storey et al., 2020) and B cells (Tangye et al., 2023). Here, reduction of *pabp2* causes an increase in the neoblast population along with a decrease in their differentiation, resulting in a defect in lineage progression toward single or multiple cell types. We tackled the effect of PABP2 deficiency in regulating differentiation using epidermal lineages as a proxy. Planarian fate diversity occurs at various stages among different lineages. A recent study demonstrated the direct specification for epidermal, intestinal, muscle, and parenchymal lineages—fate specification occurs at the neoblast stage for these lineages (King et al., 2024). Planarian epidermal lineage commitment has been extensively studied, and many markers of epidermal lineage progression have been identified (Tu et al., 2015). The upregulation of *zfp1* indicates the commitment of neoblasts to the epidermal lineage. Simultaneously, this is also evident from the maintenance of *agat1* ^+^ cells, an epidermal lineage marker. Together, these provide evidence that the stem cell self-renewal and commitment to epidermal lineages are unaffected. However, our transcriptome data revealed that the consequence of *pabp2* RNAi was observed in the latter stages of differentiation, which is evident from the drastic decrease in *zpuf 6*, and *vim 3* expression (late epidermal progeny markers) (Figure 5C). This late-stage differentiation defect could either be an effect of an intrinsically defective early progenitor or could result from the depletion of the late progenitor-specific transcript which restricted the epidermal lineage progression. These observations point to a critical role for PABP2 in regulating cellular differentiation.

In addition to the significant reduction in transcripts specific to major epidermal population, a subset of the *cathepsin* ^+^ cell type and intestinal populations is observed; we also observed a significant decrease in the *nkx1.1* ^+^ and *myoD* ^+^ cells in the *pabp2* RNAi animals. This suggests that PABP2 is crucial for the regeneration and maintenance of the muscle fibers. Here, it is likely that lesions observed in the *pabp2* RNAi animals could be due to the defective turnover of the epidermis and muscle. Embedded in the epidermis and surrounded by the muscle are the excretory units or protonephridia that regulate osmotic pressure. We did not see a depletion of protonephridial transcripts, although an abrupted protonephridial branching pattern was observed. Here, the defect at protonephridia seems like a defect caused either by an epidermal irregularity or due to an indirect effect of defective muscle cells. Several intestinal specialized neoblast markers were downregulated among *pabp2* RNAi animals. Our transcriptome shows an enrichment of *zfp1* (Figure 3E; Supplementary Tables S2, S3). *zfp1* expression is observed among intestinal neoblast expressing markers for endocytes (King et al., 2024). This could be explained as an accumulation of intrinsically defective intestinal specialized neoblasts or a defective differentiation process. Together, our study demonstrates that PABP2 is a critical regulator for stem cell differentiation toward multiple cell lineages. A detailed analysis of the impact of *pabp2* RNAi on other cell types is required, prioritizing intestinal lineages and cathepsin^+^ subtypes expressing *cela 1* and *jag 1*.

The current study provides insights into the role of PABP2 in regulating stem cell differentiation in planaria. However, it is necessary to delineate the molecular mechanism through which multifunctional PABP2 elicits its effects during regeneration. Developing PABP2 antibodies specific to *S. meditteranea* would be necessary to understand the direct interacting partners. This would enable the delineation of the mechanistic role of PABP2 in stem cell functioning. Studies have shown that mitochondrial dynamics regulate stem cell self-renewal and differentiation (Khacho and Slack, 2017; Khacho et al., 2016; Seo et al., 2018). We observed the downregulation of mitochondrial potential among *pabp2* RNAi animals (Supplementary Figure S6), suggesting a possibility that PABP2 modulates mitochondrial transcripts specific for stem cell fate determination. It is possible that PABP2 could play an inevitable role in regulating the mitochondrial transcripts necessary for stem cell differentiation. Considering the phenotype, which include lesions and subsequent lysis of animals, another aspect that requires attention is the investigation of the effect of *pabp2* RNAi on apoptosis.

Conventionally, PABP2 is believed to be ubiquitously involved in active transcription. A number of studies have analyzed its multifaceted functions, including transcriptional regulation (Bear et al., 2003), alternative polyadenylation (De Klerk et al., 2012; Jenal et al., 2012; Martine, 2012), and the hyperadenylation and decay of RNA (Bresson et al., 2013). Research on *xenopus*, mouse, and MEL (mouse erythroleukemia) cells have reported the role of poly A binding proteins in the regulation of maternal transcript processing and erythroid differentiation, thereby extrapolating its function to stem cell regulation (Cragle et al., 2019; Dai et al., 2022; Kini et al., 2014). Several molecules requisite for neoblast differentiation have been identified among *S. meditteranea: mex-3*, *CHD-4*, *apob-1, apob-2, egfr-1, nrg-1, p-66,* and *cbp-3* (Scimone et al., 2010; Wong et al., 2022; Zhu et al., 2015; Vásquez-Doorman and Petersen, 2016; Barberán et al., 2016; Fraguas et al., 2021). Our study demonstrates the requirement for PABP2 during stem cell differentiation in planaria.

## Data Availability

The datasets presented in this study can be found in online repositories. The names of the repository/repositories and accession number(s) can be found in the article/Supplementary Material.

## Supplementary material

SUPPLEMENTARY FIGURE S1 Related to Figure 1: **(A)** Phylogenetic tree showing the relatedness of PABP2 to other PABPs across different species. The PABP2 gene cluster with its homologs. Each node metadata represents bootstrap support (%). **(B)** Domain organization of PABP2 protein predicted using the conserved domain database (CDD). PABP2 protein has a single RNA recognition motif (RRM). **(C)** Multiple sequence alignment of PABP2 with PABPs of other model organisms.

SUPPLEMENTARY FIGURE S2 Related to Figure 2: **(A)** Blastema-size quantification from days 1–7 post-amputation (n = 6). **(B)** Agarose gel electrophoresis demonstrating pabp2 RNAi efficacy analyzed by PCR amplification. Actin was used as a loading control. **(C)** Timeline showing the injection regime where dsRNA was injected during the homeostasis experiment. **(D)** Homeostasis knockdown animals with lesions at the anterior region (n = 7), scale bar, 200 μm. Images were taken on the day 28 from the beginning of the dsRNA regimen. **(E)** Quantification of peripheral muscle fiber thickness (three frames from a single worm where 10 fibers were measured from each of the frames. Total fibers measured from a single worm = 30, (n = 5), ***P < 0.001. **(F)** Quantification of intensity of anti-acetylated tubulin staining for protonephridia, (n = 6), ****P* < 0.001.

SUPPLEMENTARY FIGURE S3 Related to Figure 3: **(A)** Confocal images of fluorescent *in-situ* hybridization at the regenerating surface for wi1 (scale bar, 100 μm) and the zoomed images (scale bar, 20 μm). **(B)** Quantification of the percentage of wi1+ cells, (n = 5), ***P < 0.001. **(C)** Maximum intensity projections of confocal images showing H3PS10+ cells at 2 dpa and 7 dpa among control RNAi and pabp2 RNAi, scale bar, 50 μm.

SUPPLEMENTARY FIGURE S4 Related to Figure 4: **(A)** Confocal images of entire animal showing distribution of the stem cell population (*wi1*-green) differentiating cell population (WI1- red) and immediate early progenitors (merged-yellow) (n = 6), scale bar, 100 μm.

SUPPLEMENTARY FIGURE S5 Related to Figure 5: Bar plot depicting gene expression variations. Transcripts with variations among **(A)** cathepsin^+^ cells, **(B)** pharynx, **(C)** muscle, **(D)** parapharyngeal, **(E)** neural, **(F)** ciliated neurons, and **(G)** non-ciliated neurons compared with *pabp2* RNAi and control RNAi animals during time points (as denoted by color bars within the plots) among anterior and posterior regenerating blastema. The asterisk indicates statistical significance (q < 0.05), and the dotted line on *y*-axis represents log_2_FC −0.58. Comparison is between *pabp2* RNAi *versus* control RNAi in each of the conditions denoted by the bar color within the plots. All cell-type specific transcripts with q < 0.05 in at least one of the four comparisons are included in the graph. Details about fold change and normalized read counts are provided in Supplementary Table S3.

SUPPLEMENTARY FIGURE S6 FACS analysis demonstrating reduced mitochondrial potential in *pabp2* RNAi. **(A, B)** Scatter plot from FACS analysis of control RNAi macerates stained with R123 **(A)** and control RNAi stained with R123, followed by FCCP treatment **(B)** (n = 30). **(C, D)** Scatter plot from FACS analysis of *pabp2* RNAi macerates stained with R123 **(C)** and *pabp2* RNAi macerates stained with R123, followed by FCCP treatment **(D)** (n = 30). **(E, F, G)** Graphs represent difference in mitochondrial potential among X1 **(E)**, X2 **(F)**, and Xins populations **(G)** compared with various cell populations across control and *pabp2* RNAi. The error bar is drawn from biological triplicates. **P* < 0.05.

SUPPLEMENTARY TABLE S1 Related to Figure 3: Expression values of common neoblast markers from RNA sequencing analysis at 0 dpa and 3 dpa anterior and posterior regenerating blastema.

SUPPLEMENTARY TABLE S2 Related to Figure 3: Expression values of specialized neoblast markers from RNA sequencing analysis.

SUPPLEMENTARY TABLE S3 Related to Figure 5: Expression values of differentiation markers from RNA sequencing analysis.
