# Supplementary figures and images for: Poly (A) binding protein 2 is critical for stem cell differentiation during regeneration in the planarian Schmidtea mediterranea
Source: Front Cell Dev Biol. 2024 Sep 23;12:1433142. doi: 10.3389/fcell.2024.1433142 (PMC11456742; doi:10.3389/fcell.2024.1433142)

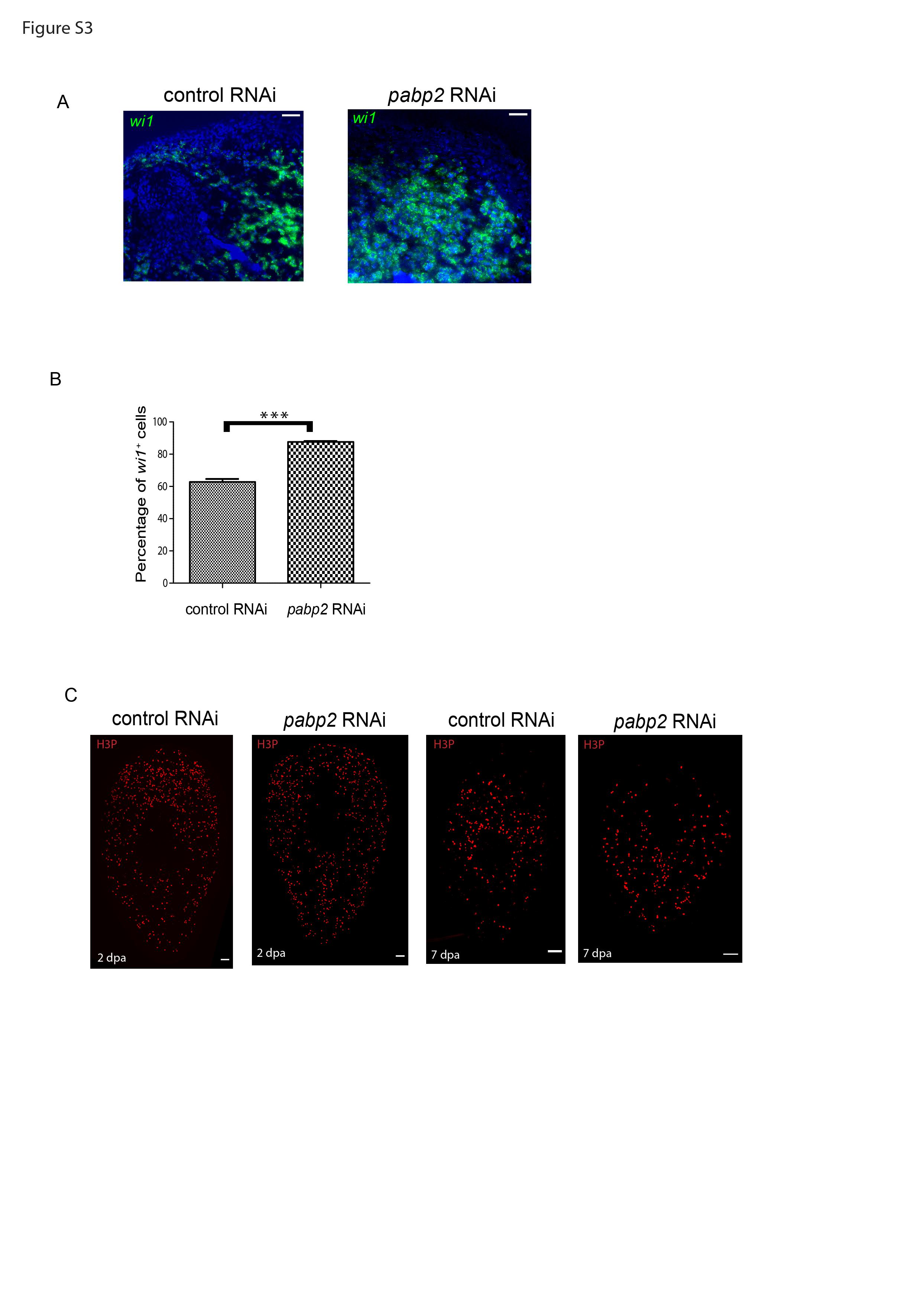

Supplement: Supplementary file 2 [file Image3.JPEG]

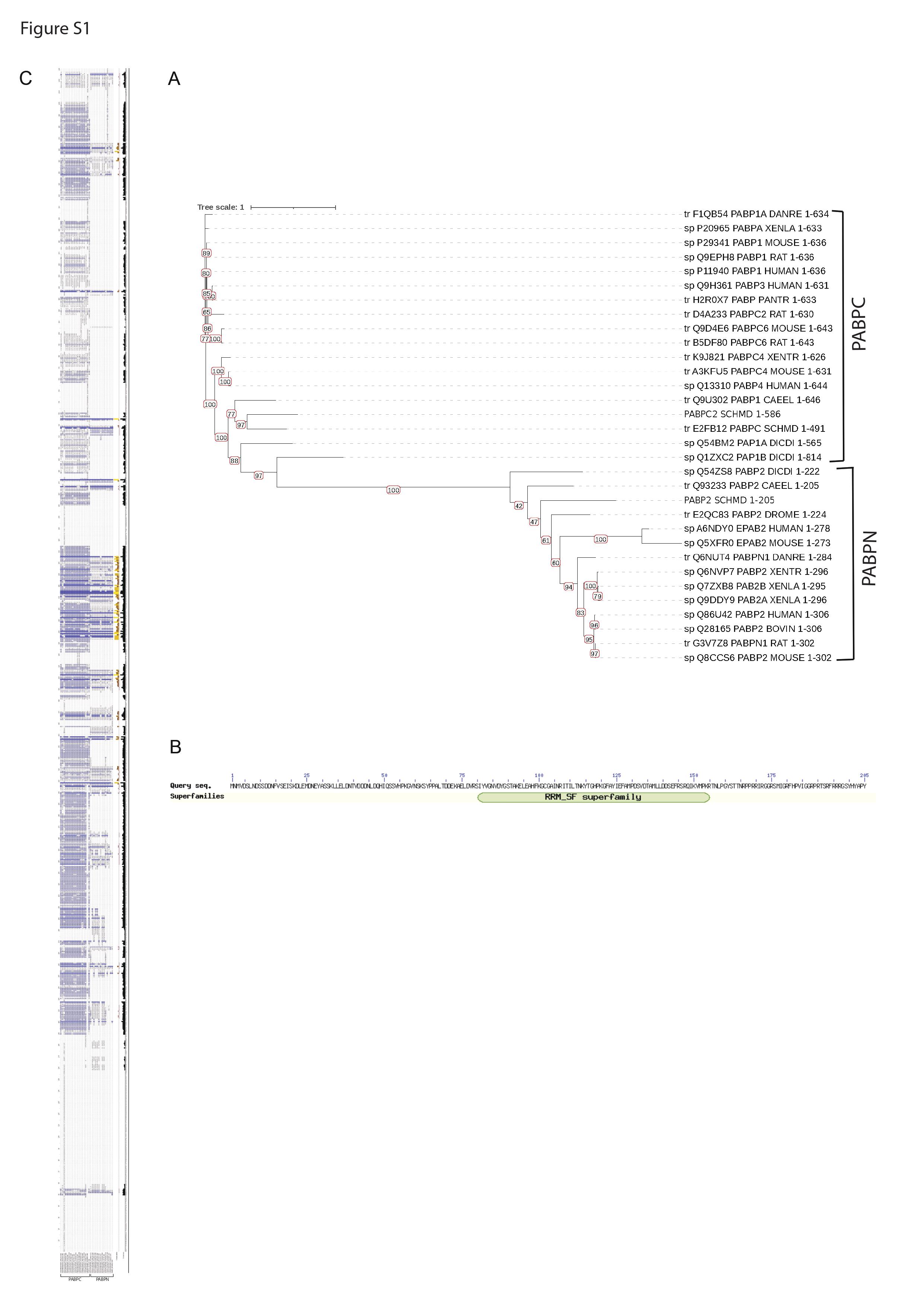

Supplement: Supplementary file 4 [file Image1.JPEG]

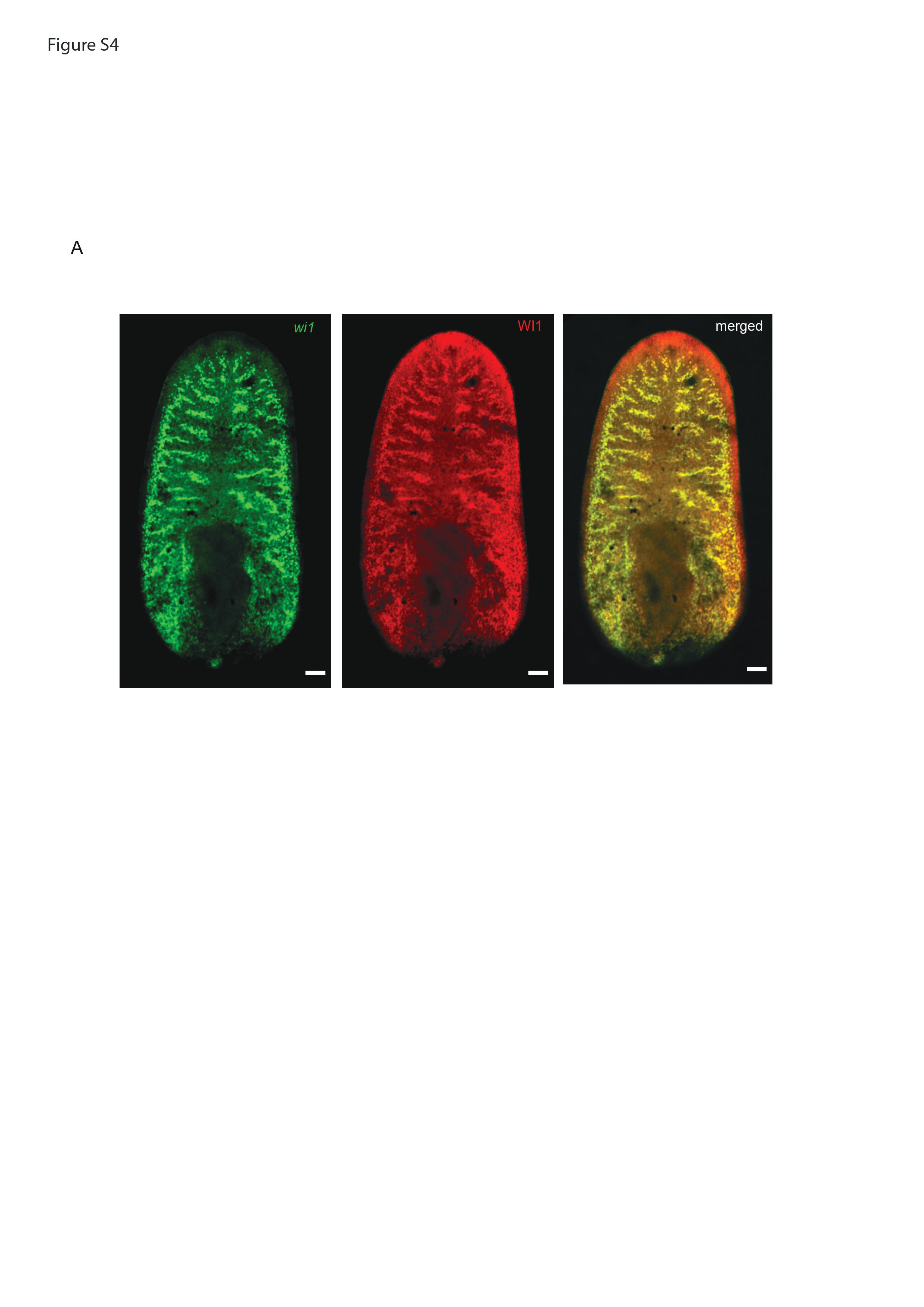

Supplement: Supplementary file 5 [file Image4.JPEG]

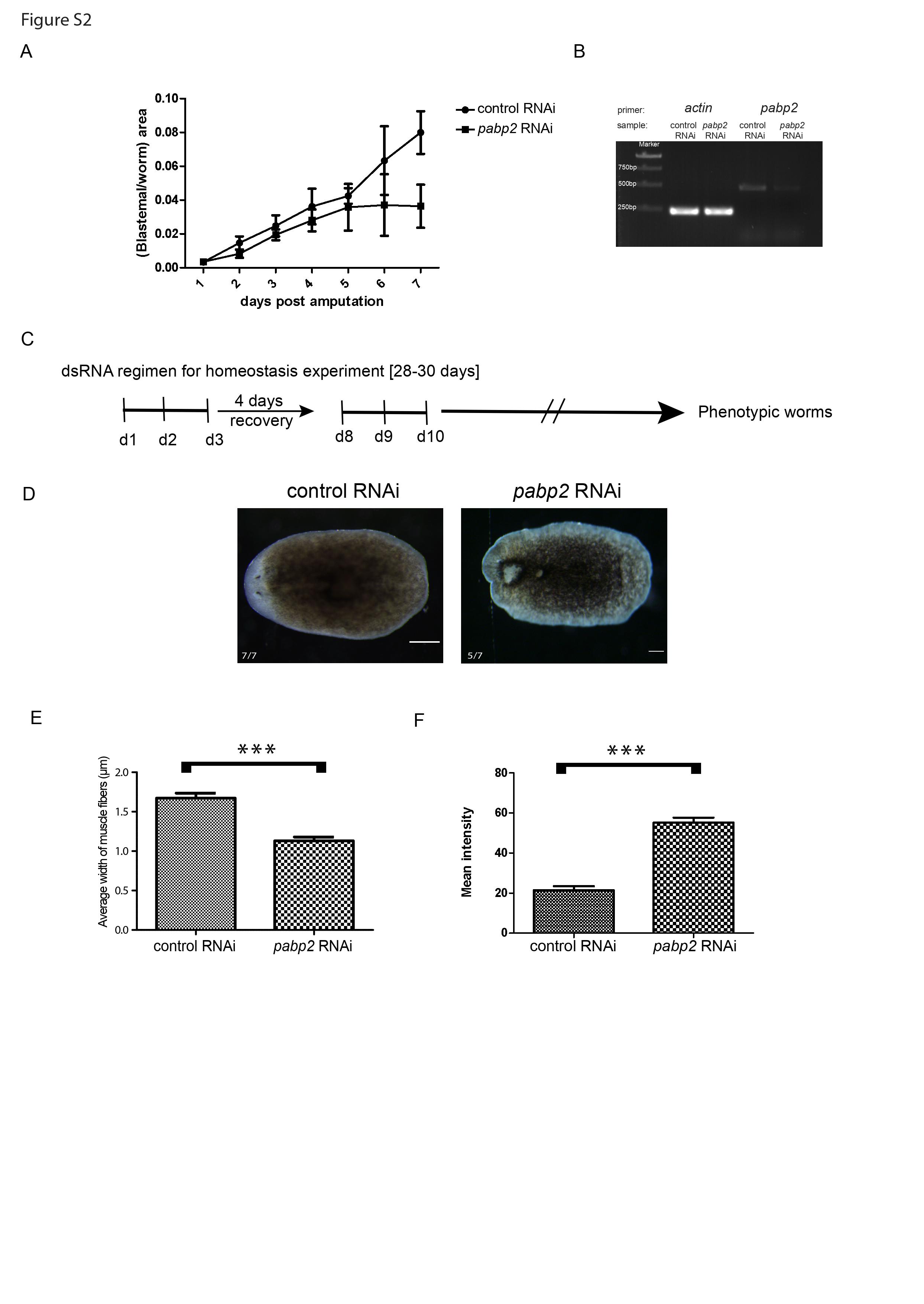

Supplement: Supplementary file 6 [file Image2.JPEG]

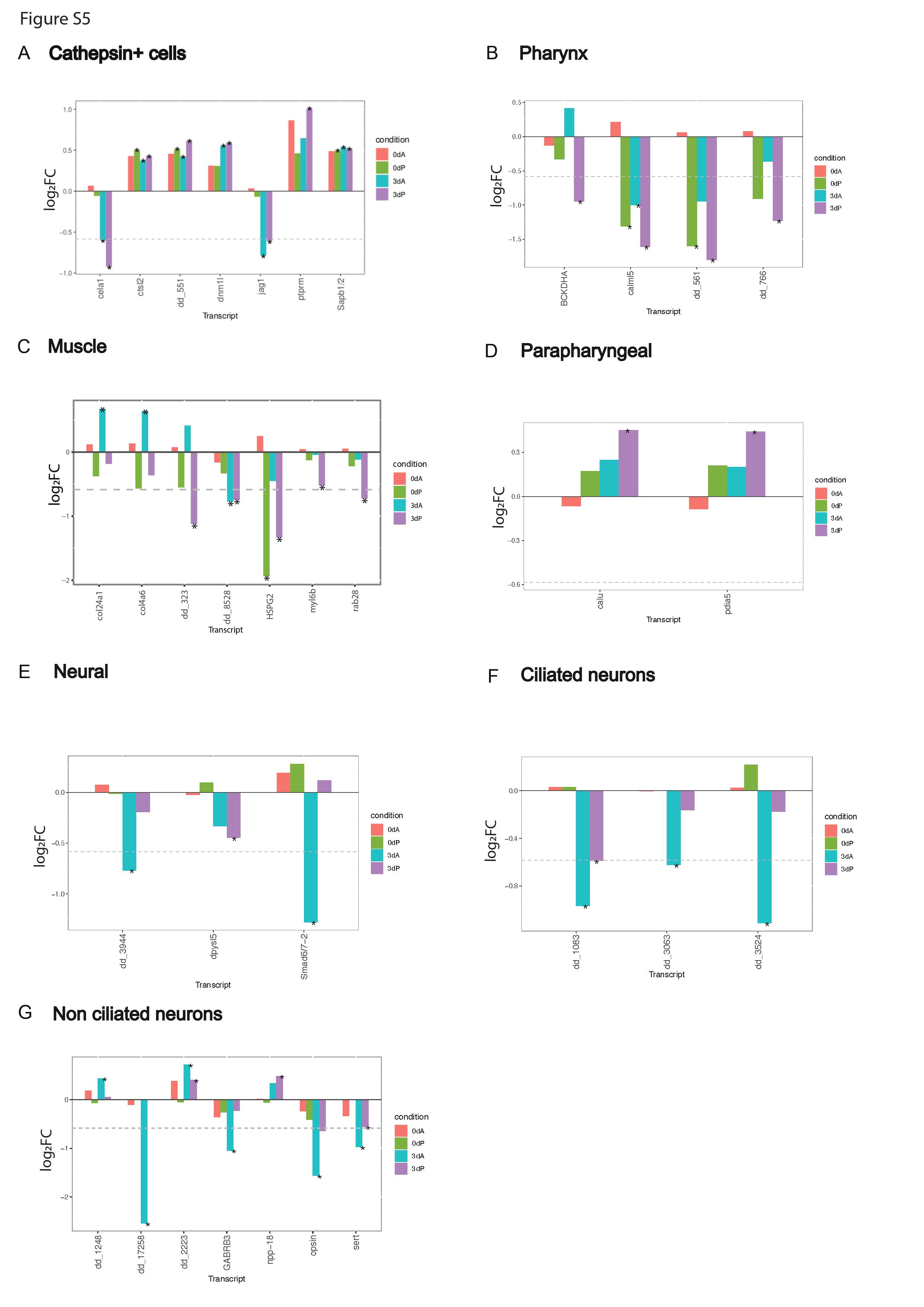

Supplement: Supplementary file 7 [file Image5.JPEG]

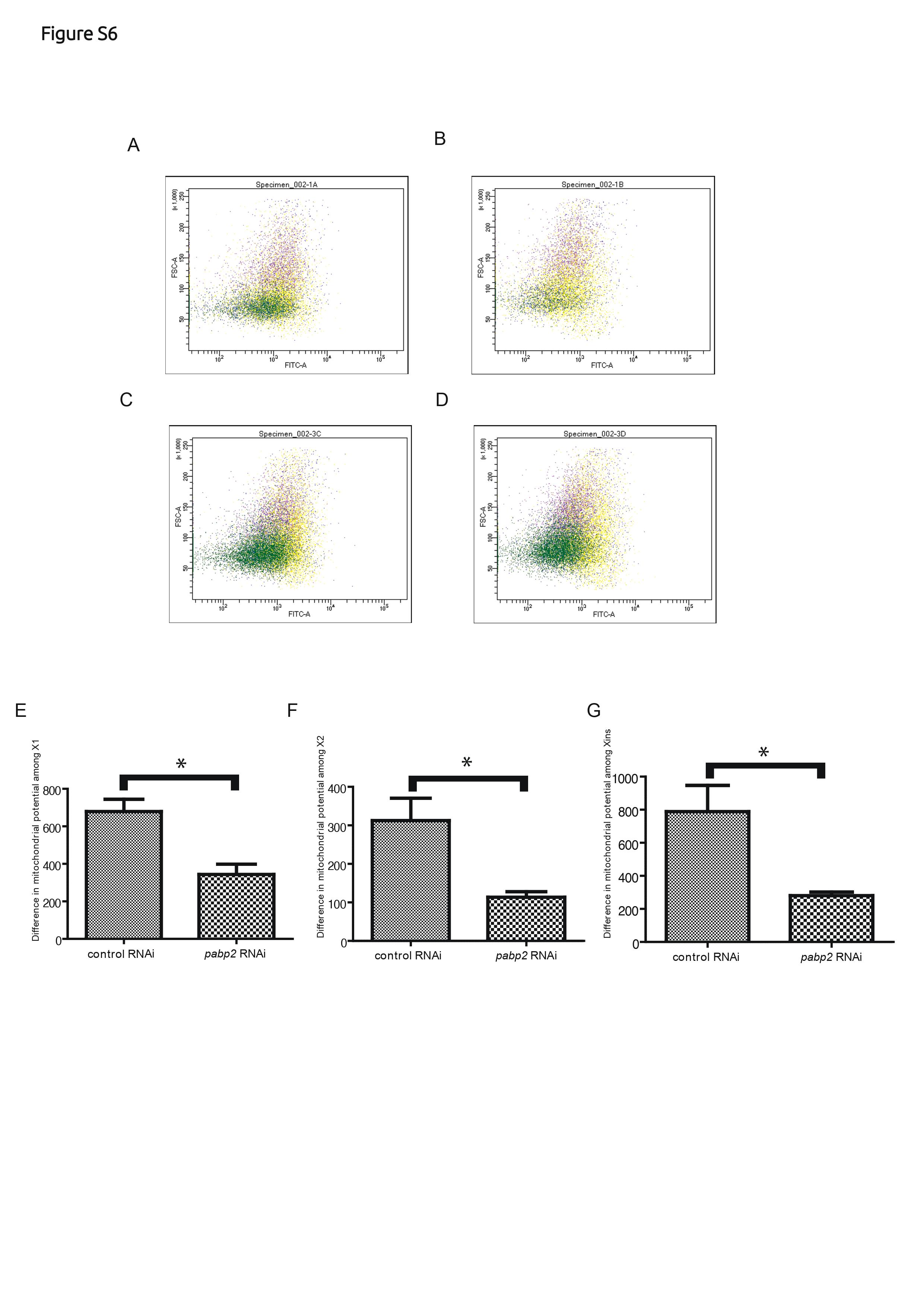

Supplement: Supplementary file 9 [file Image6.JPEG]
